# Supplementary material for: Cryo-EM structures and functional properties of CALHM channels of the human placenta
Source: eLife. 2020 May 6;9:e55853. doi: 10.7554/eLife.55853 (PMC7242029; doi:10.7554/eLife.55853)
Supplement: Supplementary file 1. [file elife-55853-supp1.docx]

| **Key Resources Table** | | | | |
| --- | --- | --- | --- | --- |
| **Reagent type (species) or resource** | **Designation** | **Source or reference** | **Identifiers** | **Additional information** |
| Antibodies | Mouse Monoclonal Cytokeratin 7 Antibody (OV-TL12/30) [Alexa Fluor^®^ 488] | Bio-Techne | Cat#NBP2-47940AF488 | Final concentration  10 µg/ml |
| Antibodies | Mouse Monoclonal Vimentin Antibody (V9) [Alexa Fluor® 647] | Bio-Techne | Cat#NBP1-97670AF647 | Final concentration  10 µg/ml |
| Antibodies | Rabbit anti-CALHM2 | Novusbio | Cat#NBP1-87936 | WB (1:500) |
| Antibodies | Rabbit anti-CALHM4 | Pineda Antibody-Service | N/A | WB (1:1000) |
| Antibodies | Rabbit anti-CALHM6 | Biorbyt | Cat#orb106933 | WB (1:500) |
| Antibodies | Goat anti-Rabbit (HRP) | Abcam | Cat#ab6721 | WB (1:5000) |
| Chemical compound, drug | HyClone HyCell TransFx-H medium | GE Healthcare | Cat#SH30939.02 |  |
| Chemical compound, drug | Phosphate Buffered Saline | Sigma | Cat#**806552** |  |
| Chemical compound, drug | L-glutamine | Sigma | Cat#G7513 |  |
| Chemical compound, drug | Penicillin-streptomycin | Sigma | Cat#P0781 |  |
| Chemical compound, drug | Fetal bovine serum | Sigma | Cat#F7524 |  |
| Chemical compound, drug | Poloxamer 188 solution | Sigma | Cat#P5556 |  |
| Chemical compound, drug | Polyethylenimine 25K MW, branched | Sigma | Cat#408727 |  |
| Chemical compound, drug | Polyethylenimine hydrochloride, 40K MW, linear | Polysciences | Cat#24765 |  |
| Chemical compound, drug | Dulbecco’s Modified Eagle’s Medium (DMEM) High glucose, pyruvate | Gibco | Cat#41966-029 |  |
| Chemical compound, drug | Valproic acid sodium salt | Sigma | Cat#P4543 |  |
| Chemical compound, drug | cOmplete, EDTA-free Protease Inhibitor Cocktail | Roche | Cat# |  |
| Chemical compound, drug | Glycol-diosgenin (GDN) | Anatrace | Cat#GDN101 |  |
| Chemical compound, drug | Lauryl Maltose Neopentyl Glycol (LMNG) | Anatrace | Cat#NG310 |  |
| Chemical compound, drug | D-desthiobiotin | Sigma | Cat#D1411 |  |
| Chemical compound, drug | Triton X-100 | Millipore Sigma | Cat#T9284 |  |
| Chemical compound, drug | Biotin | Millipore Sigma | Cat#B4501 |  |
| Chemical compound, drug | Tween 20 | Sigma | Cat#93773 |  |
| Commercial assay or kit | GoScript™ Reverse Transcriptase | Promega | Cat#A5003 |  |
| Commercial assay or kit | GoTaq^®^ qPCR Master Mix for Dye-Based Detection | Promega | Cat#A6002 |  |
| Commercial assay or kit | StrepTactin Superflow affinity resin slurry | IBA Lifesciences | Cat#2-1206-002 |  |
| Commercial assay or kit | Superose 6 10/300 GL | GE Healthcare | Cat#17-5172-01 |  |
| Commercial assay or kit | Superose 6 5/150 | GE Healthcare | Cat#29091597 |  |
| Commercial assay or kit | 200 mesh Au 1.2/1.3 cryo-EM grids | Quantifoil | Cat#N1-C14nAu20-01 |  |
| Commercial assay or kit | 200 mesh Au 0.6/1 cryo-EM grids | Quantifoil | Cat#N1-C11nAu20-01 |  |
| Commercial assay or kit | Amicon 100 kDa MWCO centrifugal filter | Millipore | Cat#UFC810096 |  |
| Commercial assay or kit | 0.22 µm Ultrafree-MCCentrifugal Filter | Millipore | Cat#UFC30GV |  |
| Commercial assay or kit | RNeasy Mini Kit | Qiagen | Cat#74104 |  |
| Commercial assay or kit | [mMESSAGE mMACHINE™ SP6 Transcription Kit](https://www.thermofisher.com/order/catalog/product/AM1340?SID=srch-srp-AM1340) | Ambion | Cat#AM1340 |  |
| Commercial assay or kit | Pierce Cell Surface Protein Isolation Kit | ThermoFisher | Cat#89881 |  |
| Commercial assay or kit | Amersham ECL Prime Western Blotting Detection Kit | GE Healthcare | RPN2232 |  |
| Cell line (human) | HEK293S GnTI- | ATCC | CRL-3022 |  |
| Cell line (human) | HEK-293T | ATCC | CRL-1573 |  |
| Recombinant DNA | Homo sapiens CALHM1 | GenScript | Accesion#NM_001001412.3 |  |
| Recombinant DNA | Homo sapiens CALHM2 | GenScript | Accesion#NM_015916.5 |  |
| Recombinant DNA | Homo sapiens CALHM3 | GenScript | Accesion#NM_001129742.2 |  |
| Recombinant DNA | Homo sapiens CALHM4 | GenScript | Accesion#NM_001366078.1 |  |
| Recombinant DNA | Homo sapiens CALHM5 | GenScript | Accesion#NM_153711.5 |  |
| Recombinant DNA | Homo sapiens CALHM6 | GenScript | Accesion#NM_001010919.3 |  |
| Recombinant DNA | pTLNX vector | Raimund Dutzler laboratory | Addgene, Cat#49032 |  |
| Recombinant DNA | Modified pcDNA 3.1 vector with C-terminal 3C protease cleavage site, Venus and Myc tags and streptavidin binding peptide | Raimund Dutzler laboratory | N/A |  |
| RNA | Antisense Cx38 | Microsynth | (Bahima et al., 2006) |  |
| Software, algorithm | ViiA™ 7 Software | Applied Biosystems | https://www.thermofisher.com/ch/en/home/life-science/pcr/real-time-pcr/real-time-pcr-instruments/viia-7-real-time-pcr-system/viia-7-software.html |  |
| Software, algorithm | SerialEM 3.5.0 | Mastronarde, 2005 | http://bio3d.colorado.edu/SerialEM/ |  |
| Software, algorithm | RELION-3.0 | Scheres, 2012 | https://www2.mrc-lmb.cam.ac.uk/relion/ |  |
| Software, algorithm | CTFFIND4.1 | Rohou and Grigorieff, 2015 | http://grigoriefflab.jan elia.org/ctf |  |
| Software, algorithm | Coot 0.8.8 | Emsley and Cowtan, 2004 | https://www2.mrc-lmb.cam.ac.uk/person al/pemsley/coot/ |  |
| Software, algorithm | PHENIX 1.14 | Adams et al., 2002 | http:// http://phenix-online.org/ |  |
| Software, algorithm | REFMAC5 | Murshudov et al., 2011 | http://www.ccpem.ac.uk/ |  |
| Software, algorithm | MSMS | Sanner et al., 1996 | http://mgltools.scripps.edu/packages/MSMS/ |  |
| Software, algorithm | DINO 0.9.4 | http://www.dino3d.org | http://www.dino3d.org |  |
| Software, algorithm | PyMOL 2.3.0 | DeLano, 2002 | https://pymol.org/2/ |  |
| Software, algorithm | Chimera 1.13.1 | Pettersen et al., 2004 | http://www.cgl.ucsf.edu/chimera/ |  |
| Software, algorithm | ChimeraX 0.7 | Goddard et al., 2018 | https://www.cgl.ucsf.edu/chimerax/ |  |
| Software, algorithm | CHARMM | Brooks et al., 1983 | https://www.charmm.org/charmm/ |  |
| Software, algorithm | Axon Clampex 10.6 | Molecular Devices | N/A |  |
| Software, algorithm | Axon Clampfit 10.6 | Molecular Devices | N/A |  |
| Software, algorithm | Prism 7 | GraphPad | https://www.graphpad.com/ |  |
