# Supplementary material for: Cryo-EM structures and functional properties of CALHM channels of the human placenta
Source: eLife. 2020 May 6;9:e55853. doi: 10.7554/eLife.55853 (PMC7242029; doi:10.7554/eLife.55853)
Supplement: Transparent reporting form [file elife-55853-transrepform.pdf]

eLIFE
1st Floor
24 Hills Road
Cambridge CB2 1JP, UK
P 01223 855340
W elifesciences.org
T @elife

eLife's transparent reporting form

We encourage authors to provide detailed information *within their submission* to facilitate the interpretation and replication of experiments. Authors can upload supporting documentation to indicate the use of appropriate reporting guidelines for health-related research (see EQUATOR Network), life science research (see the BioSharing Information Resource), or the ARRIVE guidelines for reporting work involving animal research. Where applicable, authors should refer to any relevant reporting standards documents in this form.

If you have any questions, please consult our Journal Policies and/or contact us: editorial@elifesciences.org.

Sample-size estimation

- You should state whether an appropriate sample size was computed when the study was being designed
- You should state the statistical method of sample size computation and any required assumptions
- If no explicit power analysis was used, you should describe how you decided what sample (replicate) size (number) to use

Please outline where this information can be found within the submission (e.g., sections or figure legends), or explain why this information doesn't apply to your submission:

- For expression analysis the sample size amounted to 10 for human placental tissues (Figure 1A and Figure 1—Figure Supplement 1A) and 5 for human trophoblasts isolated from placentae (Figure 1B and Figure 1—Figure Supplement 1B). In one case, the same placenta was used in both assays. Therefore, a total of 14 different placentae were screened for expression of CALHM genes.
- Electrophysiological measurements were repeated multiple times with different batches of cRNA and *X. laevis* oocytes with very similar results. The number of biological replicates ranged from 19-27 (Figure 2B-D, Figure 2—Figure Supplement 1), as indicated in Figure 2 legend. Errors are shown as standard deviations.
- The sample size for cryo-EM analysis was chosen to obtain the best possible resolution for each dataset. The detailed procedure is outlined in materials and methods section on cryo-EM data collection and image processing.

Replicates

- You should report how often each experiment was performed
- You should include a definition of biological versus technical replication
- The data obtained should be provided and sufficient information should be provided to indicate the number of independent biological and/or technical replicates
- If you encountered any outliers, you should describe how these were handled
- Criteria for exclusion/inclusion of data should be clearly stated
- High-throughput sequence data should be uploaded before submission, with a private link for reviewers provided (these are available from both GEO and ArrayExpress)

Please outline where this information can be found within the submission (e.g., sections or figure legends), or explain why this information doesn't apply to your submission:

- Number and type of replicates are indicated in each respective figure legend panel.
- For expression analysis, all replicates are biological, meaning that each measurement was done either on individual human placental tissues or individual human trophoblasts isolated from healthy term placentae. PCR amplification reactions were performed in duplicates.
- For electrophysiology experiments, all replicates are biological, meaning that each data subset was acquired from a unique oocyte injected with a corresponding cRNA.
- No obvious outliers related to the behavior of independent oocytes or protein constructs were encountered or omitted from presented data. Only outliers resulting from technical complications, such as leaky oocytes in electrophysiological recordings, were discarded.
- Presented data is in accordance with eLife's regulations and appropriate information regarding the number and type of replicates is provided in figure legends

Statistical reporting

- Statistical analysis methods should be described and justified
- Raw data should be presented in figures whenever informative to do so (typically when N per group is less than 10)
- For each experiment, you should identify the statistical tests used, exact values of N, definitions of center, methods of multiple test correction, and dispersion and precision measures (e.g., mean, median, SD, SEM, confidence intervals; and, for the major substantive results, a measure of effect size (e.g., Pearson's r, Cohen's d)
- Report exact p-values wherever possible alongside the summary statistics and 95% confidence intervals. These should be reported for all key questions and not only when the p-value is less than 0.05.

Please outline where this information can be found within the submission (e.g., sections or figure legends), or explain why this information doesn't apply to your submission:

- For expression analysis, after proving the values to be normally distributed by Shapiro-Wilk normality test, statistical evaluations were performed using paired 2-way ANOVA with Sidak's multiple comparisons test.
- For Ca^{2+} concentration-response analysis using TEVC methods, statistical significance was determined by analysis of variance by Student's t test.
- For cryo-EM maps, the resolution was estimated as the gold-standard Fourier shell correlation between two independently refined half-maps. For details, see materials and methods.

(For large datasets, or papers with a very large number of statistical tests, you may upload a single table file with tests, Ns, etc., with reference to sections in the manuscript.)

Group allocation

- Indicate how samples were allocated into experimental groups (in the case of clinical studies, please specify allocation to treatment method); if randomization was used, please also state if restricted randomization was applied

- Indicate if masking was used during group allocation, data collection and/or data analysis

Please outline where this information can be found within the submission (e.g., sections or figure legends), or explain why this information doesn't apply to your submission:

N/A

Additional data files ("source data")

- We encourage you to upload relevant additional data files, such as numerical data that are represented as a graph in a figure, or as a summary table
- Where provided, these should be in the most useful format, and they can be uploaded as "Source data" files linked to a main figure or table
- Include model definition files including the full list of parameters used
- Include code used for data analysis (e.g., R, MatLab)
- Avoid stating that data files are "available upon request"

Please indicate the figures or tables for which source data files have been provided:

No numerical data have been provided.

eLife Sciences Publications, Ltd is a limited liability non-profit non-stock corporation incorporated in the State of Delaware, USA, with company number 5030732, and is registered in the UK with company number FC030576 and branch number BR015634 at the address 1st Floor, 24 Hills Road, Cambridge CB2 1JP | August 2014
